# Myoblast and fibroblast derived small extracellular vesicles differentially affect myoblast migration dynamics

**DOI:** 10.1007/s10974-025-09705-y

**Published:** 2025-08-18

**Authors:** Kyle N. Hagemann, Rhys S. McColl, Jason A. C. Lovett, Celia Snyman, Kathryn H. Myburgh

**Affiliations:** Department of Physiological Sciences, Stellenbosch University, Stellenbosch, 7600 South Africa

**Keywords:** Extracellular vesicles, Myoblasts, Fibroblasts, Intercellular communication, Uptake, Migration

## Abstract

Muscle injury activates satellite cells and fibroblasts, with extracellular vesicles (EVs) mediating the related intercellular communication. The influence of EVs released by either cell type on recipient cell behaviour is still unclear. This study investigated the uptake and effects of EVs derived from C2C12 myoblasts (myo-EVs) and L929 fibroblasts (fibro-EVs) on proliferating myoblasts. Both cell lines were cultured in media largely depleted of FBS-derived EVs. Myo-EVs and fibro-EVs isolated from conditioned media were characterised using conventional methods. Effects of these EVs on myoblast function were assessed via PKH67-labelled EV uptake, proliferation, scratch closure, leading front migration rate and individual cell trajectories and western blot analysis for MyoD and Myogenin. Myoblasts preferentially internalised myo-EVs at 5 h (myo-EVs: 3.41 ± 1.34 vs fibro-EVs: 1.25 ± 1.13 puncta per cell) and 48 h (myo-EVs 16.55 ± 12.60 vs fibro-EVs 9.67 ± 4.88) (both p < 0.05). Under proliferative EV-depleted conditions, added EVs did not change myoblast proliferation. However, the elevated expression of Myogenin indicating a subtle shift toward differentiation. Myo-EVs increased myoblast migration rate into a scratch, compared to controls (13.77 ± 1.7 vs 11.08 ± 2.23 µm/h, p < 0.01), but had no effect under conditions of FBS EV depletion. On the other hand, fibro-EVs increased the speed of individual cells, but negatively affected leading front migration due to impaired myoblast persistence. These findings highlight the importance of cell-specific EV-mediated communication in muscle regeneration. Further, tissue explants used to generate cell-specific EVs for treatment should be free of contaminating cell types.

## Introduction

The regeneration of skeletal muscle after injury relies on a multitude of processes, from initial stem cell activation to myoblast fusion and differentiation into myotubes and myofibres. Control mechanisms for these processes include a transient inflammatory response, and a highly coordinated interplay of intracellular myogenic regulatory factors (MRFs) (Forterre et al. 2014a). Muscle healing requires a diverse set of cell types within the muscle niche where satellite cells, the myoblast progenitors crucial for tissue repair, are located. These cell populations include satellite cells, fibroblasts that produce extracellular matrix (ECM) for structural support and immune cells that promote inflammation in reaction to damage and endothelial cells in the vasculature (Rubenstein et al. 2020). Communication between these different cell types relies on local paracrine interactions or systemic signalling to induce activation of satellite cells (Rubenstein et al. 2020; Ceafalan et al. 2014). Vesicles secreted from source cells that contain biologically active molecules are increasingly shown to be part of this paracrine communication system (Avalos and Forsthoefel 2022).

Extracellular vesicles (EVs) are a heterogeneous group of lipid bilayer-enclosed nano-sized vesicles that are typically categorised based on their size, into small vesicles or exosomes (30–150 nm), microvesicles or medium vesicles (100–1000 nm), and apoptotic bodies (> 1 µm), each with distinct mechanisms of biogenesis and secretion (Van Niel et al. 2018; El Andaloussi et al. 2013; Yáñez-Mó et al. 2015). Since a specific size cut-off for exosomes is not universally agreed upon, small EVs may follow the continuum of size to approximately 200 nm, as stated in the MISEV 2023 guidelines (Welsh et al. 2024). Small EVs are derived from the endosomal system and subsequently secreted from the cell. These small vesicles protect and transport a wide range of bioactive molecules including proteins, lipids, nucleic acids, cytokines, and growth factors (Daaboul et al. 2016). Emerging roles for small EVs in intercellular communication have become more apparent (Avalos and Forsthoefel 2022).

EVs secreted from most of the cells in the muscle microenvironment play roles in intercellular communication that are becoming increasingly prominent. Byun et al. (2021) and Nakamura et al. (2015) showed that cultured mesenchymal stem cells (MSCs) release small EVs that promoted myofiber regeneration both in vitro and in vivo by stimulating gene expression of MRFs, including myoblast determination protein 1 (MyoD) and Myogenin. MSC-derived EVs also reduced the in vivo expression of inflammatory TGFbeta in a contraction-induced muscle injury in a mouse model (Iyer et al. 2020). Recent studies have highlighted the role of EVs secreted from in vitro human skeletal myoblasts (Choi et al. 2016) and C2C12 myoblasts (myo-EVs) in muscle regeneration (Aswad et al. 2016; Forterre et al. 2014b; Guescini et al. 2017; Ji et al. 2022; Kim et al. 2018). The application of such cell-derived EVs to an injured mouse model provided biochemical cues for skeletal muscle regeneration leading to increased stem cell-related Pax7 gene expression, elevated myofibre-related myosin heavy chain and desmin expression and inhibition of fibrosis (Ji et al. 2022; Choi et al. 2016). Also, proteins in the membrane or on the periphery of EVs released from electrically stimulated C2C12 myotubes increased mitochondrial oxygen consumption rates and ATP production in recipient myoblasts to maximal levels (Obi et al. 2025).

Muscle-related fibroblasts are the main contributor to ECM production in muscle (Chapman et al. 2016). Interactive paracrine control between skeletal myoblasts and ECM secretion by fibroblasts has been alluded to (Fry et al. 2017). EVs derived from normal fibroblasts (fibro-EVs) have been implicated in various regenerative processes, including fibroblast proliferation and migration (Oh et al. 2021). EVs secreted by fibroblasts in vitro facilitate wound healing and angiogenesis by endothelial cells in the skin (Oh et al. 2021). Depending on the tissue of origin though, fibroblast EVs have different protein cargo which may change the composition of the ECM and affect tissue architecture (Yeung et al. 2020). Disease may affect cargo carried in EVs, and the influence that such cargo may have on recipient cells. As an example, in patients suffering from Collagen VI-related muscular dystrophy, EVs released from primary cultured fibroblasts significantly altered motility patterns of control fibroblasts in a migration assay (Badosa et al. 2023). Still, specific functions of fibro-EVs in the context of the skeletal muscle niche remain poorly understood. All these findings suggest co-operative roles for EVs derived from both satellite cells and fibroblasts in the muscle niche to manage both the ECM environment and the process of myoblast differentiation (Chapman et al. 2016; Fry et al. 2017).

Recent evidence has shown, though, that foetal bovine serum (FBS) added to regular cell culture media contain EVs and that such EVs may control the very activities in C2C12 myoblasts and other cell types that are under investigation (Aswad et al. 2016; Sawada et al. 2022; Urzì et al. 2022). EVs isolated from FBS inhibited C2C12 myoblast differentiation (Sawada et al. 2022; Urzì et al. 2022). Proliferation of C2C12 cells was reduced in EV-depleted media and during subsequent differentiation, despite the increase in myogenin, myotube formation was impaired and could not later be restored on addition of FBS-derived EVs (Aswad et al. 2016). This indicates that EVs contained in FBS may mimic the paracrine communication between myoblasts and myotubes.

We hypothesised that EVs secreted from two niche-related cell types may differentially influence myoblast activities related to repair and growth of skeletal muscle as part of a paracrine intercellular communication system. This study aimed to explore the roles of EVs in the regulation of skeletal muscle regeneration. We put particular emphasis on the contributions of EVs derived from L929 fibroblasts and C2C12 myoblasts in modulating processes important for the regenerative response, including proliferation and aspects of migration. Due to recent evidence that FBS-derived EVs affect myoblast activity, for this study FBS was depleted of EVs prior to its addition to complete culture media. Cell-type specific EVs were isolated from the media conditioned by myoblasts (myo-EVs) or fibroblasts (fibro-EVs). Crucially, the uptake of these two types of EVs into myoblasts was assessed in a culture environment free of exogeneous EVs.

## Materials and methods

### Production of extracellular vesicle-depleted foetal bovine serum

EVs were removed from FBS (EV-free FBS) (Gibco, Ref. 10,500–064, USA) using differential dUC. Briefly, FBS was ultracentrifuged at 100 000 × g for 16 h at 4 °C (Beckman-Coulter, OptimatmL-80-XP ultracentrifuge, type 70Ti fixed-angle rotor, USA) in 29.9 mL polypropylene UC tubes (OptiSeal, Beckman-Coulter, Ref. 361,625, USA). This speed and duration have been previously reported to be optimal for EV removal (Théry et al. 2006). Once completed, 20 mL of the upper supernatant was removed using a hypodermic needle and syringe and was then filtered through a 0.22 μm filter. This EV-depleted FBS was then aliquoted into 15 mL falcon tubes and stored at −20 °C.

### Cell culture

The immortalised mouse myoblast C2C12 cell line and the mouse fibroblast L929 cell line were cultured in normal growth media (NGM) which consisted of DMEM with high glucose and without glutamine (Gibco, Ref. 11,960–044, USA), supplemented with 2% (v/v) L-glutamine (Gibco, Ref. 25,030–081, USA), 2% (v/v) Penicillin–Streptomycin (Gibco, Ref. 15,140–122, USA) and 10% FBS. All cells were initially cultured in T75 flasks with NGM and incubated at 37 °C with 5% CO2. Cells were grown to 80% confluency, passaged, and media was replenished every 2 days. Experiments were performed using passages between 11 and 16 for C2C12 cells, and before 26 for L929 cells. EV-depleted media (EV-DM) was prepared by replacing FBS with 10% (v/v) EV-depleted FBS.

### Conditioned media production

For conditioned media production, C2C12 myoblasts and L929 fibroblasts were seeded in T175 flasks at 8 × 105 and 15 × 105 cells respectively. Cells were cultured in 30 mL NGM for 24 h, after which the media was removed, the cells were washed with PBS and 30 mL of fresh EV-DM was added. Conditioned media was harvested after 48 h and stored at −20 °C.

### EV isolation from conditioned media using differential ultracentrifugation

Conditioned media (30 mL) collected from C2C12 and L929 cultures were subsequently thawed and centrifuged at 300 × g for 10 min to remove cells and the supernatant was centrifuged at 2000 × g for 20 min to remove any dead cells. The resulting supernatant was then transferred to 29.9 mL polypropylene UC tubes (OptiSeal, Beckman-Coulter, Ref. 361,625, USA) and the total volume was made up to 29 mL with PBS. These samples were ultracentrifuged at 10 000 × g for 30 min to remove large vesicles, after which the supernatant was transferred into new UC tubes, topped with PBS, and then ultracentrifuged again at 100 000 × g for 2 h. This final step has been shown to be the optimal speed and time for final small EV isolation (Théry et al. 2006). The final supernatant was discarded, and each pellet was resuspended in 400 μL of freshly filtered (0.22 μm) PBS. Eight such resuspended samples originally harvested from T175 flasks were pooled to ensure consistent composition for downstream purposes. A small volume of this pooled sample was used to assess the particle concentration prior to freezing using NTA, and the rest of the sample was aliquoted and stored as individual treatment samples.

### Nanoparticle tracking analysis

NTA was used to determine the particle concentration and size distribution or mode (Nanosight LM10, Malvern Panalytical, UK) of pooled samples is isolated from myoblast and fibroblast conditioned media prior to freezing. The volume retained from the pooled sample was diluted 25 × in particle-free PBS. The temperature was set manually, the camera level was set to 16, and the screen gain was set to 10. For video analysis, the detection threshold was set to 6. Three 30 s videos were analysed (3 technical repeats of one pooled sample). Values corresponding to particle size and concentration were determined by the software.

### Protein concentration of the EV samples

A Micro bicinchoninic acid (BCA) kit (Thermofisher, USA) was used for protein quantification in EV samples isolated from myoblast and fibroblast conditioned media.

### Scanning electron microscopy

Carbon-coated, 200-mesh copper SEM grids (Ted Pella Inc. Ref. 01840, USA) were glow discharged (10 s; 10 mA) using a sputter coater (Leica EM ACE200, Leica Microsystems, Germany) and 10 μl of the pooled resuspended EV sample was incubated on the grids for 10 min. The grids were then washed with dH20, dabbed with filter paper (Whatman, UK), and incubated on freshly filtered 2% uranyl acetate (Fisher Scientific, Ref 18–607-644) for 2 min. Grids were dabbed, washed with dH20 again, and allowed to dry at room temperature for 10 min. High-resolution SEM analysis was performed at 22 kV (ThermoFisher Apreo Serial Block Face FESEM, Thermofisher, USA).

### Western blotting

As part of the characterisation of the pooled myo-EV and fibro-EV samples collected from respective conditioned media, western blotting was performed on these EV samples, as well as on a myoblast cell lysate that served as a control sample. The myoblast cell lysates were prepared by incubation in a final concentration of 1X RIPA buffer (5 mM Tris–HCl, 15 mM NaCl, 0.1% Triton X-100, 0.025% sodium deoxycholate, 0.1 mM EDTA, pH 7.4) containing both protease inhibitors (Roche, Ref. 04693116001, Switzerland) and phosphatase inhibitors (Roche, Ref. 04906837001, Switzerland) for 10 min on ice. Samples were then stored at −20 °C.

Total protein in the samples was denatured with 4X Laemmli sample buffer (62.5 mM Tris–HCL, pH 6.4, 2% (w/v) SDS, 10% (v/v) glycerol, 0.005% (w/v) bromophenol blue, 10% (v/v) β-mercaptoethanol) and then heated to 95 °C for 5 min. EV lysate (30 μg per well) and cell lysates (15 μg per well) and a pre-stained protein ladder (PageRuler™, Thermofisher, Ref. 26,620, USA) were loaded and separated on a 12.5% SDS-PAGE gel. The gel was run at 120 V in running buffer and was subsequently transferred to a PVDF membrane using a semi-dry transfer system (Trans-blot Turbo, Bio-Rad, USA). The transfer was done at 25 V and 2.5 mA for 20 min in transfer buffer (25 mM Tris, 192 mM glycine, 20% (v/v) methanol, pH 8.3). The membrane was subsequently blocked in 5% (v/v) fat-free milk in TBS-T buffer (0.15 M NaCl, 0.02 M Tris–HCl, 0.1% Tween® 20, pH 7.6) for 1 h. The membrane was washed in TBS-T and initially probed with a primary antibody against Follistatin diluted in TBS-T containing 1% (w/v) BSA at 4 °C overnight, and then for 4 h at room temperature, on a roller mixer. The membrane was washed multiple times with TBS-T before treatment with an HRP-linked secondary antibody in TBS-T with 1% (w/v) BSA for 1 h at room temperature on a roller. The membrane was then again washed with TBS-T and incubated with Enhanced chemiluminescence (ECL) substrate (Thermo Scientific, cat.34095, USA) and a ChemiDocTM MP Imaging System (Bio-Rad, USA) was used to visualise protein bands. After imaging, the membrane was stripped of all antibodies with a mild stripping buffer containing ß-mercaptoethanol (Kar et al. 2015), washed extensively and re-probed in a consecutive fashion, first for the presence of Alix and then for TSG101. Finally, densitometry analysis of the blot images was carried out using commercially available software (Image Lab version 6.1, BioRad, USA) and the relative protein band intensities were determined and normalised against Glyceraldehyde 3-phosphate dehydrogenase (GAPDH). All antibody information can be found in Table 1.

**Table 1:** Antibodies and dilutions used for Western blotting

Primary antibodies	Cat. No	Dilution
Alix Mouse Monoclonal Antibody (3A9)	sc-53538	1:500
Follistatin Rabbit Polyclonal Antibody	Ref. ab47941	1:500
TSG101 Mouse Monoclonal Antibody (C-2)	Ref. sc-7964	1:500
MyoD1 Rabbit Monoclonal Antibody (D8G3)	Ref. 13,812	1:400
Myogenin Mouse Monoclonal Antibody (F5D)	Ref. 14–5643-82	1:500
GAPDH Rabbit Polyclonal Antibody	Ref. ab9485	1:1000
Secondary antibodies		
Anti-mouse IgG HRP-linked Antibody	Ref. 07/2015	1:1000
Anti-rabbit IgG HRP-linked	Ref. 02/2016	1:1000

The effect on the levels of two myogenic regulatory factors (MyoD and Myogenin) in EV-treated myoblast lysates were also analysed using western blots. GAPDH was used as an internal loading control. Briefly, C2C12 myoblast cultures were exposed to myo-Evs or fibro-EVs for 48 h under proliferative conditions. Control cell lysates were prepared from C2C12 myoblast cells grown in NGM for 48 h. After EV treatment the growth medium was removed, and the wells were washed three times with PBS. After removal of PBS, RIPA buffer was added to each well. The cells were detached into the buffer using a scraper (NEST Biotech, cat. 710,001) and the cell suspensions from all the wells were collected and transferred into 1.5 mL tubes (Eppendorf, USA) and incubated on ice for 1 h. The suspensions were then sonicated (3 × 2 s pulses at 30% power rate) (Intelligent Ultrasonic Processor, Bueno Biotech, China) and centrifuged at 16 000 × g for 10 min. The supernatant was collected, and protein concentrations were determined using a BCA assay (Thermofisher, USA) following the manufacturer's protocol. Lysates were then stored at −80 °C. Samples were denatured, resolved on a 12.5% SDS-PAGE gel and transferred to PVDF membrane. The membranes were probed with MyoD and Myogenin antibodies and HRP-linked secondary antibodies, as described above.

### EV membrane labelling and uptake into myoblasts

In order to visualise EVs for uptake studies, myo- and fibro-EVs were labelled with 4 μL PKH67 lipophilic dye for 5 min (Merck, Ref. MINI67-KT, USA), quenched with 1% BSA to bind any free dye, and then ultracentrifuged at 100 000 × g for 70 min to further remove any unbound dye. Filtered PBS which went through the same labelling process as EVs, was included as a dye control. This control measure confirmed that all free unbound dye was removed during the quenching and ultracentrifugation steps.

Sterile coverslips were placed in 24-well plates and incubated with entactin-collagen IV-laminin (ECL) at 37 °C for 2 h. C2C12 myoblasts were seeded in NGM at 7500 cells/cm^2^ and allowed to adhere. After 6 h cells were washed and treated with the PKH67-labelled EVs in EV-DM as a baseline and again at the 24 h time point. EV uptake by myoblast cells was measured after either a 5 h or 48 h period, using confocal microscopy.

For confocal microscopy, cells were fixed with 10% formalin (Leica Biosystems, Ref. 3800604EG, USA) for 15 min, washed with PBS, and permeabilised with 0.1% Triton X-100 for 15 min. Cells were then incubated with an f-actin stain (Alexa FluorTM Plus 647 Phalloidin, Thermofisher, Ref. A30107, USA) for 1 h at room temperature. Cells were washed and incubated with Hoechst for 5 min. Coverslips were mounted on glass slides and the cells were imaged using a confocal microscope (Carl Zeiss LSM780 with ELYRA S.1 Super-resolution platform, Carl Zeiss, Germany). The total number of cells, number of puncta and cells containing puncta were counted in each of 5 fields of view and the average number of puncta per cell per field of view was presented in a graph format.

### Myoblast proliferation analysis

C2C12 myoblasts were seeded in 6-well plates at a density of 7500 cells per cm^2^ and grown in 2.5 mL of NGM for 6 h to promote adherence. The cell monolayer was subsequently washed once with DMEM, and the media was replaced with 2.5 mL of either NGM or EV-DM. At this time point, cells grown in EV-DM were also treated with EVs derived from either C2C12 myoblasts (myo-EV) or L929 fibroblasts (fibro-EV) at a concentration of 2 × 10^8^ particles/mL, or an equal volume of sterilised and filtered PBS as a vehicle (NGM control). After 24 h, a 50% media replacement was done, as well as a second treatment for the treatment groups with the same concentration of EVs (2 × 10^8^ particles/mL). Cells were cultured for a further 24 h.

Images were captured using an in-incubator imaging system (Olympus Provi CM20, Olympus, Japan). An automated image analysis protocol was set up. A standard experimental protocol was run, with the automated analysis imaging every 3 h for 48 h. The same analysis protocol was used for all experimental repeats. Setting up an analysis protocol involves training the Provi to identify cells. Images from low, medium, and high confluency periods were used, and what was considered a cell and what was considered background was indicated to the Provi. Training was performed and modified until cells were recognised at all confluency levels. Secondly, a cell count threshold was set for sensitivity. This was done manually, determining a threshold where manual counting corresponds to automated counting. The threshold was then kept the same for all experiments. Cell counts were acquired every three hours. Proliferation data acquired from all fields of view from the Provi CM20 was averaged to provide an overall mean value per repeat. The results were expressed as a ratio of growth over time and was used to compare proliferative capacity for each condition. Data was expressed as mean ± SEM. Significant differences were determined using a one-way ANOVA with a Tukey post-hoc test. **** p < 0.0001. n = 5.

### Scratch closure analysis of myoblasts

C2C12 myoblasts were seeded in 6-well plates at 20 000 cells per cm^2^ and grown in NGM for 24 h until 90% confluency. A standardised scratch was then performed in the cell monolayer using a commercially available scratch device (SPLScar™, SPL Lifesciences, Ref. 201,907, South Korea). Any debris was washed away with DMEM and either NGM or EV-DM was added to the wells. Two groups cultured in EV-DM were treated with either myo- or fibro-EVs (2 × 10^8^ particles per mL per treatment). Scratch closure was assessed over 10 h using an in-incubator imaging bright field microscope (Provi CM20), After 5 h, 50% of the media was replaced with fresh media containing the same EV concentrations as the initial treatment. Images were recorded every 20 min over the course of the 10 h experiment. The scale was set to 0.4600 pixels/µm. The variance was set to 15, the threshold value set to 50, and the percentage of saturated pixels was set to 0.001%. The closure of the scratch was assessed in three ways: firstly, the area of the scratch; secondly, the migration of the leading front and thirdly, the migration pattern of individual cells.

The dynamic % scratch closure over the 10 h duration was automatically quantified using the Wound Healing Size Tool plugin for ImageJ (Suarez-Arnedo et al. 2020). This algorithm uses pixel values in a stack of binarised images to distinguish the scratch (area devoid of cells) from the surrounding cell-containing area and then determines the area of each scratch by using calibrated pixel size. Both the dynamic % scratch closure over the time course and the scratch areas at the final 10 h time point were plotted.

The rate of migration of the cells forming the leading edge into the scratch area was assessed. The difference in scratch width was measured as Euclidean distances (linear across the midline of the scratch) at the 0 h and 10 h time points. Four averaged values over 5 fields of view at each time point were used (n = 20). The rate of migration of the cell front was calculated as the difference between the distances at these two time points, using the formula below. To take into account the migration of cells from opposite sides of the scratch, the data were halved to reflect the migration front from one side of the scratch only.

Leading front migration rate: $\frac{{Width}_{T0}-{Width}_{T10}}{10 hours}$ T0 = original scratch width at 0 h.

T10 = final scratch width at 10 h.

In addition, individual cell tracking was performed using a second plugin (MtrackJ plugin, ImageJ). Ten individual cells from each side of the scratch (n = 20) were tracked in each of 5 fields of view; n = 4 repeats; for a total of 400. Image sequences were loaded in ImageJ with the scale set to 0.4600 pixels/µm. A grid overlayed on the image was used to select a total of twenty cells from both sides of the scratch cells closest to the designated lines were chosen. Scatter plots were drawn using the ImageJ Migration. The coordinates provided by the MtrackJ plugin were normalised to the starting point of the cell, for the first coordinates to be (0, 0). Using these normalised coordinates, the accumulated, speed, linear distances and persistence were determined over the 10 h period. First, data for the 20 cells in each field of view were average. Graphs were created using GraphPad Prism 9 software and represent the means of 5 fields of view for 4 repeats (n = 20). The speed and persistence of individually tracked cells were calculated as follows:

$$\text {Speed:} {\frac{\text {Total}\, \text {Distance}\,\text {Travelled}} {\text {Time} \left(10 \text {h}\right)}}$$

$$\text {Persistence}:\frac{\text {Linear}\, \text {Distance}}{\text {Total}\, \text {Distance}\,\text{Travelled}}$$

### Statistical analysis

All the data were presented as mean ± standard error of the mean unless stated otherwise. The statistical analysis performed was either an unpaired student’s t-test or a one-way ANOVA with a Tukey post-hoc test (GraphPad Prism version 9.2.0 (322), GraphPad Software, Inc., USA). A P-value < 0.05 was considered significant. Analysis of EV uptake into myoblasts was performed via a 2-way mixed-model ANOVA and an LSD post hoc, performed in Statistica. Because of the large number of 0 puncta in the control condition, the data were log10-transformed before this statistical analysis.

## Results

### Extracellular vesicle characterisation

**Fig. 1:**
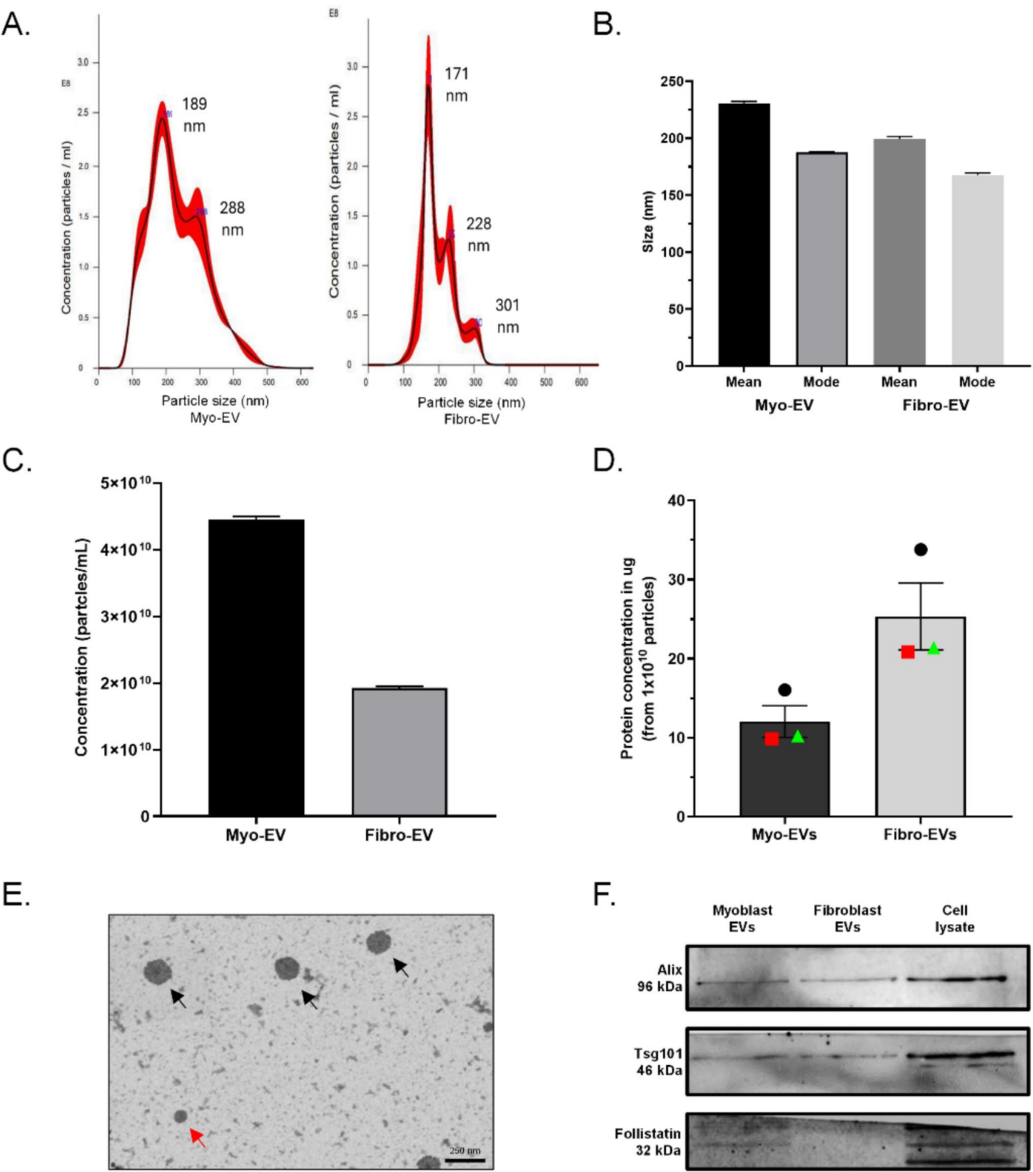
Characterisation of pooled small extracellular vesicles derived from myoblasts and fibroblasts. **A** NTA results represented the concentration and size distribution of the pooled EV samples that were used in later treatment studies. **B** Vesicle concentration of EVs collected from either myoblast or fibroblast conditioned media. **C** Mean and mode size of vesicles derived from the two different cell types. Data are expressed as mean ± SD (n = 1, pooled sample; with 3 technical repeats). **D** Protein concentration from the two EV isolates was determined using a micro-BCA kit. Data is shown as µg protein per 1 × 10^10^ particles. Data expressed as mean ± SEM. **E** SEM micrograph of EVs shows particles smaller than 200 nm in diameter (arrows). SEM scale bar = 250 nm. **F** Western blots of EV markers Alix and TSG101, and Follistatin as a cell marker relevant to myoblasts

In this study, C2C12 myoblast- and L929 fibroblast-derived EVs isolated from conditioned media were characterised using nanoparticle tracking analysis (NTA), electrophoresis and scanning electron microscopy (SEM) and the protein concentrations were quantified (micro-BCA). The size distribution plot of myo-EVs showed a highest peak concentration of EVs at 189 nm. There was a second lower concentration peak at 288 nm (Fig. 1A). The fibro-EVs showed the highest concentration peak at 171 nm. Two smaller populations of particles were evident with the second peak at 228 nm and a low concentration peak at 301 nm in diameter (Fig. 1A). The resultant mean (p < 0.0001) and the mode (p < 0.0008) size of myo-EVs was larger than that of fibro-EVs (Fig. 1B). However, the mode (most commonly occurring particle size) for both EV types was below the 200 nm level, confirming the exosome or small EV status. The mean particle concentration of the myo-EVs was significantly higher (2.5 x) than that of the fibro-EVs (p < 0.0001) (Fig. 1C). Despite this lower concentration, the fibro-EV sample had a protein concentration that was about double that of the myo-EV sample (Fig. 1D). Electron microscopy confirmed a population of nano-vesicles smaller than 200 nm (Fig. 1E).

Our NTA results contained some evidence of medium-sized EVs, although these were at the lower end of a size range (2nd mode < 300 nm) for medium EVs of 200–1000 nm (Lischnig et al. 2022). Considering the NTA mode values (highest frequency in distribution– the particle size or size range most commonly found in the distribution was 189 nm myo-EV, and 171 nm for fibro-EVs) and SEM images we concluded that our protocol likely eliminated large vesicles, and that the bulk of the vesicle populations isolated fell into the small EV category (generally smaller than 200 nm). The low ratio of larger vesicles present in our samples were considered to be at numbers low enough to not influence our findings. Western blotting analysis of the EV isolates from both cell lines revealed the presence of recognised EV markers of endosomal origin; Alix facilitates the sorting of tetraspanins into exosomes via multi-vesicular bodies (Han et al. 2022), and TSG101 is a core ESCRT component involved in the budding and release of exosomes (Zhang et al. 2019). TSG101 was expressed in myoblast cell lysate but with no, or low abundance in EVs (Lautaoja et al. 2020). Follistatin was present in small amounts in the myo-EVs and to a much greater extent in the cell lysate obtained from myoblasts grown in NGM but was absent from the fibro-EVs (Fig. 1F). Follistatin was selected for assay due to its specificity in muscle (Fodor et al. 2017).

### Extracellular vesicle uptake in myoblasts

**Fig. 2:**
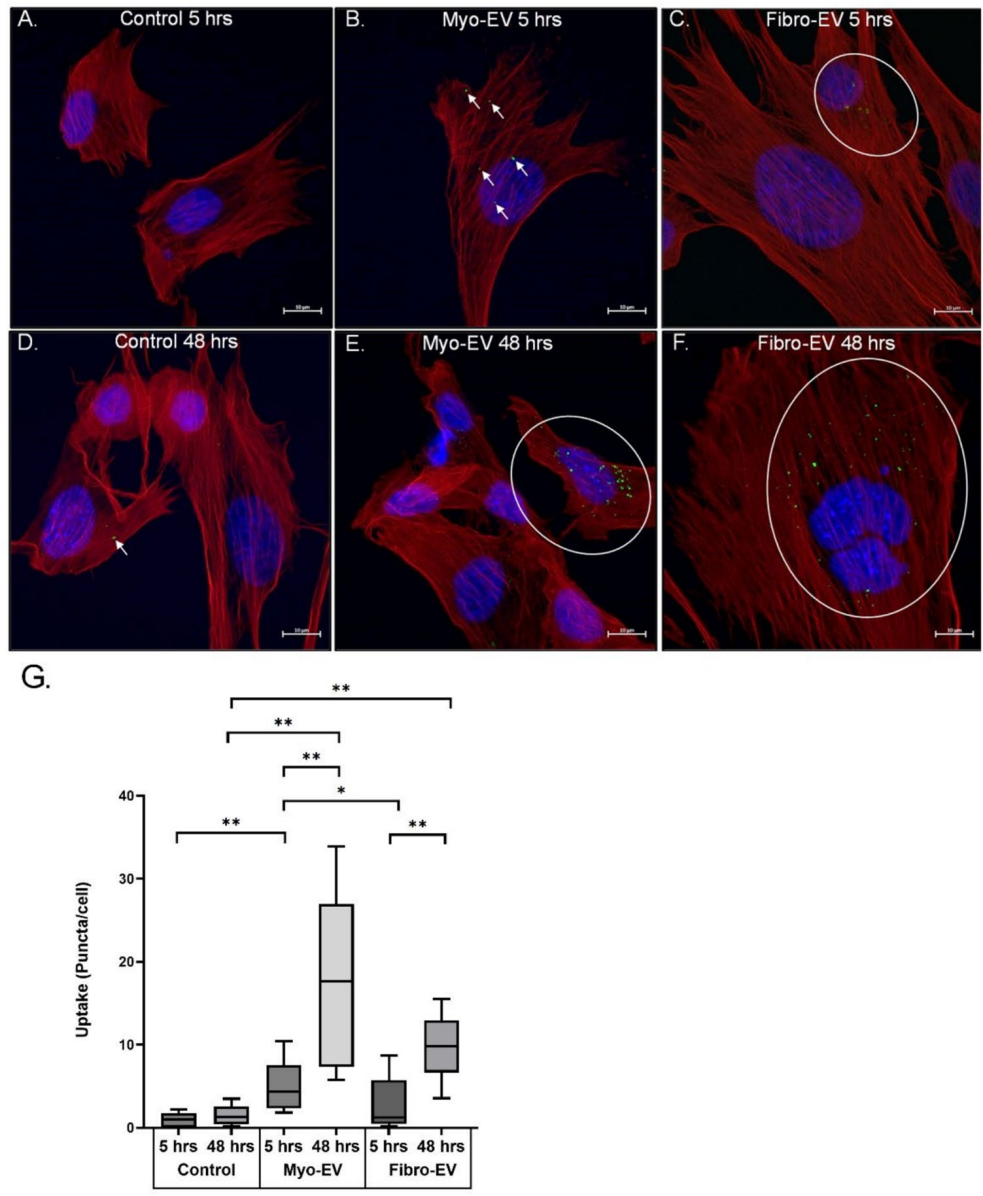
Uptake of myoblast- and fibroblast-derived EVs by myoblasts. **A**, **D** Representative images of the control group at 5 h and 48 h after exposure to PBS/PKH67 dye. Myoblasts were treated with PKH67-labelled EVs (green) at 0 h and at 24 h, and confocal microscopy images were taken at 5 and 48 h (24 h after the second treatment). Myo- and fibro-EV uptake was confirmed by green puncta within the cytoplasm at 5 h (**B**, **C**) and 48 h (**E**, **F**). Nuclei were labelled with Hoechst (blue) and f-actin was labelled with Phalloidin (red). Scale bar = 10 µm. **G** EV uptake was quantified by counting the number of green puncta at 5 and 48 h after EV treatment. Data expressed as mean ± SEM (n = 4). The data were log10-transformed before statistical analysis by a 2-way mixed-model ANOVA, and an LSD post hoc test (Statistica). ** p < 0.01, * p < 0.05. Significant differences were determined by one-way ANOVA with a Tukey post-hoc test. * p < 0.05

A PKH67 membrane dye allowed both myo- and fibro-EVs (1 × 10^8^ particles per well) taken up into myoblasts to be visualised and counted after 5 h (Fig. 2A-C) and after 48 h (Fig. 2D-F) using confocal microscopy images. Phalloidin-stained actin and Hoechst-stained nuclei facilitated with the localisation of EVs within the cells.

Even though the counts were very low in the control cells, expression of the data in a log10-transformed format provided a clear indication of the statistically significant increase in myo-EV uptake by myoblasts after 5 h, compared to both the 5 h control and the 5 h fibro-EV group (myo-EVs 3.41 ± 1.34 puncta per cell vs fibro-EVs 1.25 ± 1.13, p < 0.05). After 48 h the increase in uptake of myo-EVs was significantly elevated compared to the 48 h control and also the 5 h myo-EV data (Fig. 2G; p < 0.01). On the other hand, although the uptake of fibro-EVs after 5 h was only slightly elevated, after 48 h the fibro-EV uptake was significantly higher than both the 48 h control and the fibro-EV data at 5 h (both p < 0.01). At the 48-h time point the mean number of puncta taken up into the myoblasts was not significantly different for the EVs derived from each cell type (myo-EVs 16.55 ± 12.60 vs fibro-EVs 9.67 ± 4.88), possibly due to the variability of the number of puncta per cell at this point. For EVs from both cell types there was significant increase in uptake between the 5- and 48-h time points.

### The effects of myo- and fibro-EVs on myoblast proliferation and MRF expression

**Fig. 3:**
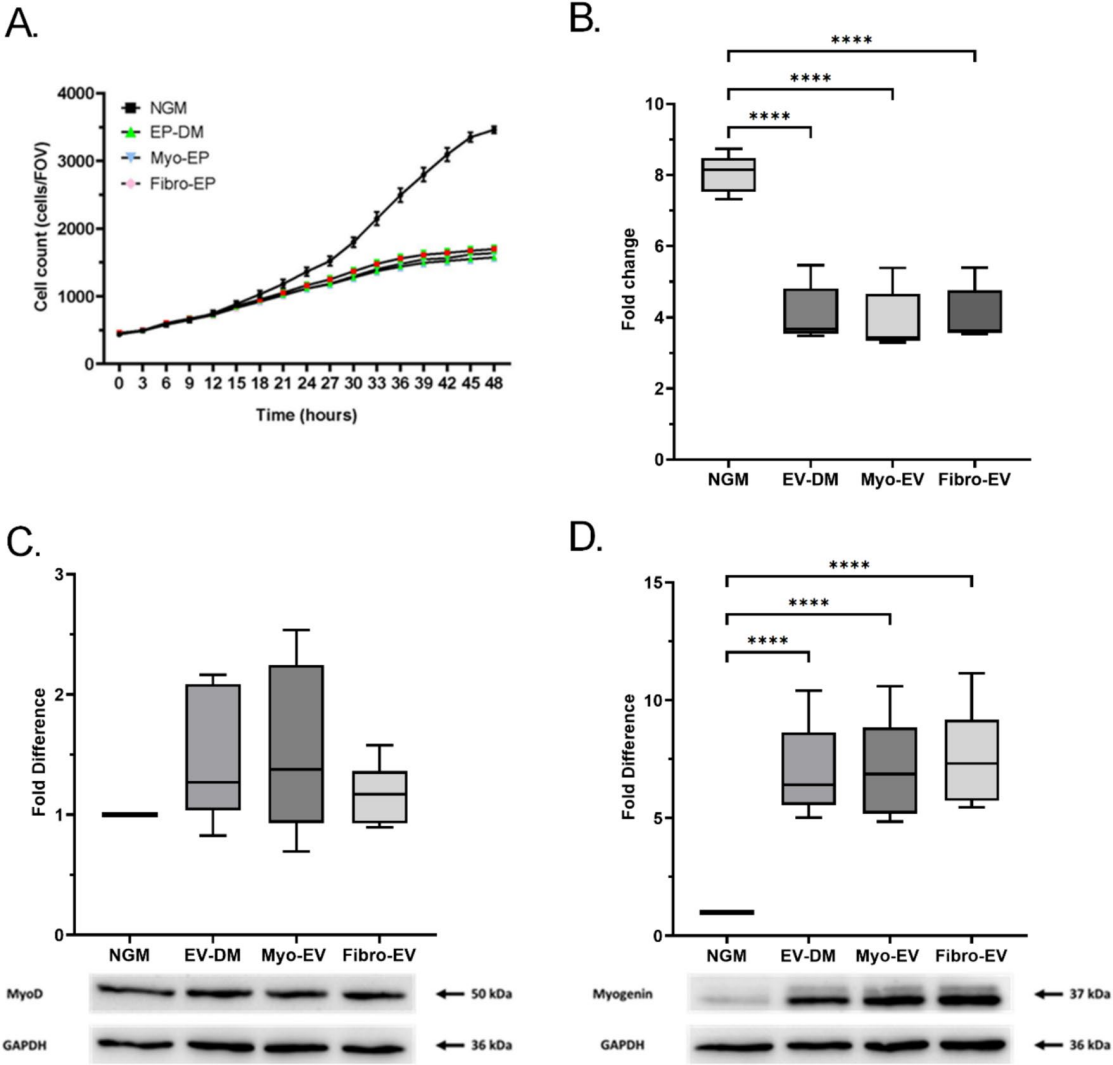
Proliferation and MRF expression of myoblasts after treatment with EVs. **A** Myoblasts were treated with myoblast and fibroblast-derived small EVs (5 × 10^8^ particles per well) at 0 h and 24 h, and proliferation was monitored using an in-incubator imaging system. Cell counts were acquired every three hours. **B** The fold change in cell counts after 48 h (n = 5). **C** MyoD and **D** Myogenin expression was analysed after 48 h. Densitometry analysis of the blot images was carried out using the BioRad Image Lab and the relative protein band intensities were determined using GAPDH as a loading control. Data expressed as mean ± SEM. Significant differences were determined using a one-way ANOVA with a Tukey post-hoc test. **** p < 0.0001

Next, in order to investigate the effect of EVs secreted from different cell types into the microenvironment of the myoblast, EVs previously collected from myoblast and fibroblast conditioned media were added to myoblast cultures. Proliferation of myoblasts in NGM was compared to that in EV-DM and after the addition of myo-EVs and fibro-EVs to EV-DM. Over the culture period there was no difference in the proliferation rate (a ratio of cell number over time) of the EV-treated cells in comparison to the EV-DM (Fig. 3A), but after 24 h all mean values were significantly lower than in the control NGM indicating an effect of FBS depletion. At the 48-h time point the fold changes in cell numbers were significantly lower for both treatment groups and EV-DM compared to NGM (n = 5; p < 0.0001) (Fig. 3B). These results showed that the reduction in proliferation in the EV-treated cells was merely due to the depletion of EVs from the culture medium and not related to the addition of EVs.

It has been shown that FBS used in NGM contains exosomes that inhibit C2C12 differentiation (Sawada et al. 2022). It is also well established that reduction in serum concentration in the culture media leads to C2C12 cells entering the differentiation phase (Lesmana et al. 2019). Therefore, it was of interest to determine if the expression of differentiation-related regulatory proteins, MyoD and Myogenin was higher in the proliferating myoblasts cultured in media depleted of EVs. This media contained the regular 10% FBS but as completed using FBS depleted of 85.3% of the EVs (EV-DM). For the Western blot analysis data were presented as a fold change relative to NGM. There was no difference in the fold change relative to the NGM of MyoD across all groups at 48 h, (NGM: 1.0; EV-DM: 1.5 ± 0.22; myo-EV: 1.5 ± 0.28; fibro-EV: 1.2 ± 0.10) although substantial variations in results were observed within the EV-DM and myo-EV groups (Fig. 3C). Mean levels of Myogenin, however, were significantly increased for the EV-DM, myo-EV, and fibro-EV groups when compared to the NGM group (NGM: 1.0; EV-DM: 7.0 ± 0.80; myo-EV: 7.1 ± 0.88; fibro-EV: 7.6 ± 0.84, p < 0.0001) (Fig. 3D). These variances in MyoD levels and increased levels of Myogenin were also clear in the EV-DM, which indicated that these results were most possibly due to the reduction in EV concentration in the media depleted EVs and not as a result of the added cell-type specific EVs. It did confirm that reduction of EV concentration in the FBS added to culture media initiates differentiation in a myoblast culture.

### The effect of myoblast- and fibroblast-derived EVs on myoblast migration

**Fig. 4:**
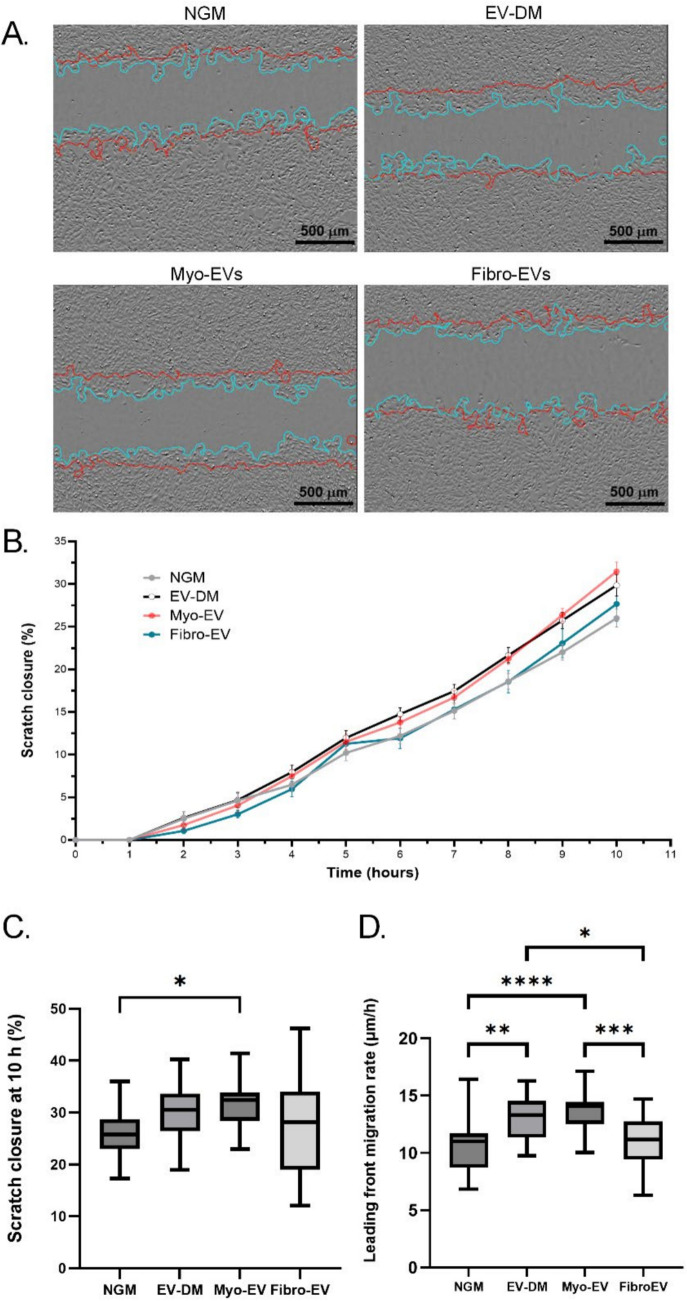
Myoblast scratch closure following treatment with small EVs derived from either myoblasts or fibroblasts. In vitro scratch assays were used to analyse the migration and mobility of myoblasts following EV treatments. Myoblasts were treated with EVs (treatment: 2 × 10^8^ particles per mL) at 0 h and 5 h after creation of the scratch and migration was monitored over a 10 h period. **A** Representative brightfield images of scratches after 10 h. The red lines indicate the scratch border at 0 h. The blue outlines show the scratch border at 10 h post scratch. **B** Percentage scratch closure at each hour between 0 and 10 h. **C** Total scratch closure at 10 h. **D** Rate of scratch closure over 10 h. One-way ANOVAs along with the Tukey post-hoc test were used to determine statistical significance between scratch closure at values 10 h and the closure rates. * p < 0.05; ** p < 0.01

A scratch assay was employed to assess the effect of myo-EVs and fibro-EVs on the migration of myoblasts. Media containing EV-depleted FBS (EV-DM) was used during the assay, alongside the control media (NGM) to ensure that any effects noted would be related only to the added EVs. Outlines of the scratch edges at the start and end of the closure were overlayed over the final scratch image (Fig. 4A). Scratch closure as measured by the reduction in the area of the initial scratch over the 10 h period is presented as a line graph that showed little variance in the closure pattern of all groups for the first 5 h of the assay, whereafter the groups began to diverge (Fig. 4B). At the 10 h time point it was only the myo-EV group that closed the scratch significantly faster than the control culture in NGM (p < 0.05). However, the myo-EV group did not differ from EV-DM or fibro-EV groups. Extensive variation was observed, especially from the fibro-EV culture (Fig. 4C).

Next, the rate of cumulative migration of the leading front cells was assessed. Such migration is representative of the linear displacement of the leading front from the initial scratch outline to the outline at the end of the treatment period and was calculated by assessing the change in width of a scratch at these time points. The EV-DM culture migrated significantly faster than the NGM cultures (13.12 ± 2.04 vs 10.62 ± 2.30 um/h; p < 0.01) (Fig. 4D). Similarly, the myo-EV culture moved significantly faster and differed to a greater extent from NGM (13.77 ± 1.70 vs 10.62 ± 2.30 um/h; p < 0.0001). In contrast, the addition of fibro-EVs limited closure rate to the same level as the NGM control (11.08 ± 2.23 vs 10.62 ± 2.30 um/h), suggesting the fibro-EVs had a possible similar inhibitory effect on myoblast migration as the EVs contained in FBS. Also, the fibro-EVs culture migrated significantly slower than the myo-EV-treated cultures (11.08 ± 2.23 vs 13.77 ± 1.70 um/h p < 0.001) and the EV-DM (13.12 ± 2.04 um/h; p < 0.05). These results also confirmed that experimental EV treatment of cultures should be performed in a medium containing FBS depleted of EVs, rather than to the medium that contained complete FBS.

**Fig. 5:**
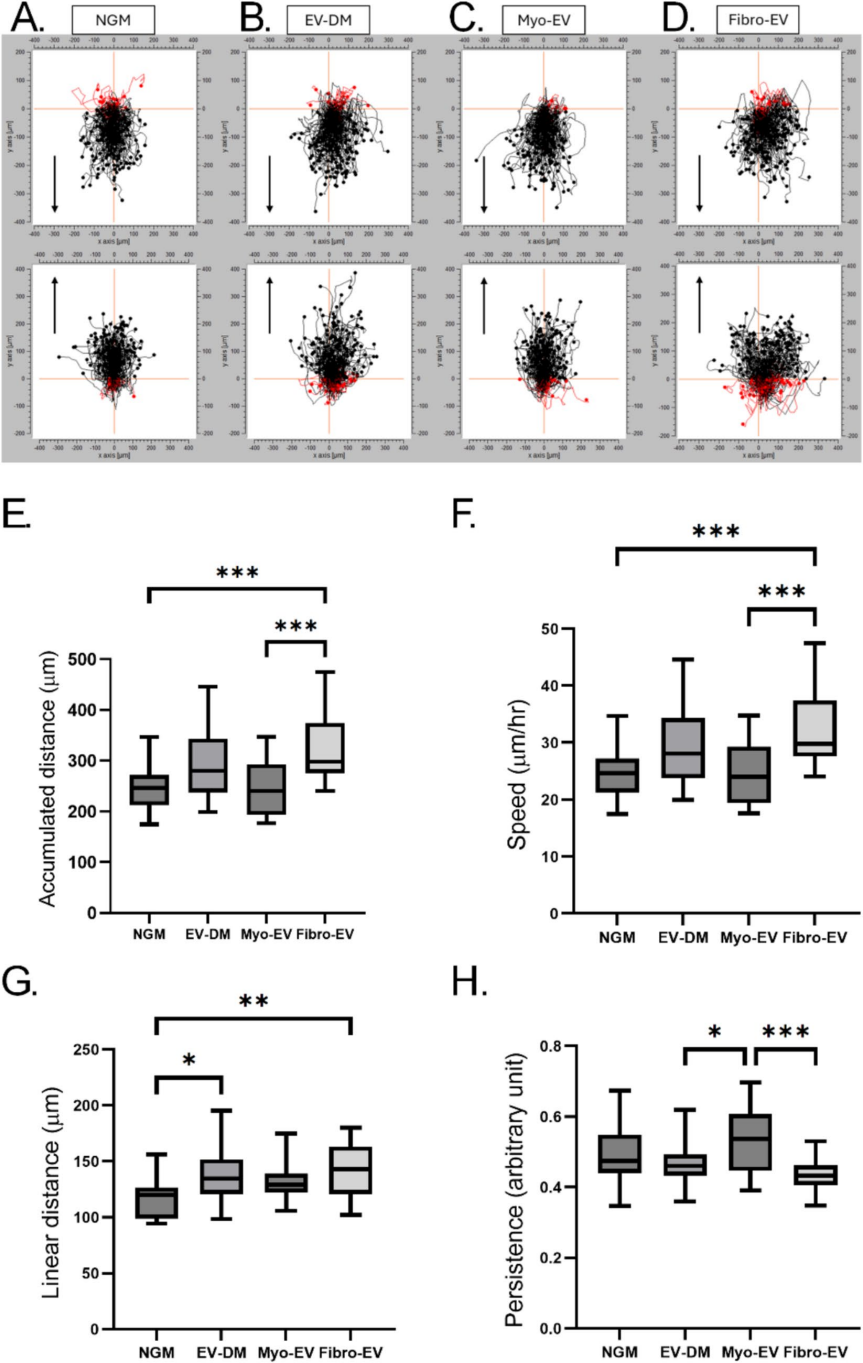
Tracking of individual myoblasts migrating into a scratch. **A–D** Migration plots were generated for each treatment group, monitoring cells from both sides of the scratch (total 200). Arrows indicate direction of migration into the scratch. From tracking individual cells the following 4 descriptors of migration could be determined: **E** accumulated distance, **F** speed, **G** linear distance and **H** persistence

To further understand the extent to which individual cells contributed to the general closure of a scratch, especially on addition of fibro-EVs, scatter plots were generated of migrating myoblasts using the MtrackJ ImageJ plugin (Fig. 5A). Parameters obtained that may explain the patterns of cell migration were analysed. The accumulated or total distances travelled by each cell along its trajectory (Fig. 5E) and the mean values of the linear distances (displacement from starting to at time 0 h to end point at time 10 h) (Fig. 5F) were determined and mapped. Using these data the speed (total distance over time) (Fig. 5G) and persistence (ratio of the total distance to the linear displacement) (Fig. 5H) were calculated and plotted. Persistence refers to the adherence of the cell trajectory to a general direction of movement, regardless of a final goal, or in the absence of a chemotactic gradient, as was the case in this assay. The lower this value, the more random and meandering the movement, resulting in low persistence.

For persistence, a value closer to 1 indicates a straighter route travelled. Data expressed as mean ± SEM. Significant differences were determined using a one-way ANOVA with a Tukey post-hoc test. * = p < 0.05; ** = p < 0.01.

Firstly, considering addition of cell-specific EVs, myoblasts cultured in the presence of fibro-EVs had significantly longer accumulated distances and hence higher speed compared to the addition of myo-EVs (Fig. 5E and F, both p < 0.001). In contrast, despite this lower speed and accumulated distance in myo-EV culture compared to the fibro-EV culture, the linear displacement of the myo-EVs was more similar to their accumulated distance, hence their directional persistence was better (Fig. 5G and H). Secondly, comparing the two cell-specific EV groups to the EV-DM group, there were no differences in accumulated distance or speed, whereas the persistence was somewhat lower than the myo-EV group (p < 0.05). myoblasts cultured in the presence of myo-EVs was significantly better in comparison to both the fibro-EVs and EV-DM, p < 0.001 and p < 0.05 respectively). Since these three groups were all cultured in conditions depleted of FBS EVs, the findings reflect the effects of the added cell-specific EVs. Finally, when considering differences from NGM, it stands out that the fibro-EV group had significantly higher accumulated distance and speed (p < 0.01), although the persistence did not differ between these two conditions.

When interpreting all the data obtained for the scratch assay, individual cell tracking data reflected a few differences for myo-EV cultures that hinted at possibly contributing to the improved overall % scratch closure that was evident only in this group. In contrast, the high variability of % scratch closure for fibro-EV culture (Fig. 4D) could be assigned to low directional persistence in the rapidly migrating myoblasts (Figure H).

## Discussion

The interconnected regulation of cell fate between cells inhabiting the stem cell micro-environment including myofibers, satellite cells, and immune cells has previously been reviewed (Porcu et al. 2024; Van Niel et al. 2018; Vumbaca et al. 2021). Small EVs and their cargo are an integral part of this complex and multicellular crosstalk.

Secreted EVs were shown to influence cells of similar origin. EVs were collected from differentiated C2C12 myotubes after electrical stimulation that resulted in chronic contraction. Myoblasts treated with these EVs showed improved mitochondrial biogenesis (Obi et al. 2025). In an in vivo study exosomes derived from myoblasts improved repair and regeneration of injured mouse skeletal muscle (Ji et al. 2022).

EVs from one cell type may influence a different cell type. Consider, for example, the crosstalk between myoblasts and fibro–adipogenic progenitors (FAPs). In a study using primary cultures from mouse skeletal muscle, EVs isolated from myoblasts inhibited adipogenesis of FAPs. Reciprocally, the FAPs released EVs that stimulated differentiation in myoblasts (Yu et al. 2024). Also, EVs secreted from cultured human myotubes (HSkM) improved the fusion index of human adipose-derived stem cells (HASCs) (Choi et al. 2016). EVs may also act in pathological conditions. The EVs secreted from stress-induced senescent primary myoblasts inhibited proliferation in an endothelial cell line (Hettinger et al. 2021).

In all these studies the definitive outcome was related to the specific EV cargo. For example, myoblast EVs that contained IL1α and IL1β (Yu et al. 2024) or miR-206-3p (Vumbaca et al. 2021) inhibited adipogenesis in FAPs, whereas FAP EVs containing miR-127–3p stimulated differentiation in myoblasts (Yu et al. 2024).

Work published by Vumbaca et al. (2021) discussed the EV-mediated crosstalk between cells found in the muscle niche. In that study, EVs were isolated from injured mouse muscle and effects on satellite cells and fibro-adipogenic cells were established in vitro. In this study, to ensure the origin of EVs was not from a mixed muscle sample but rather from a single cell type, we used EVs secreted by either myoblasts or matrix-related fibroblasts in vitro to determine their individual effect on C2C12 myoblasts*.* The EVs isolated from myoblasts and fibroblasts were characterised via reference markers, including Alix and TSG101, which confirmed the endosomal origin of both EV samples (Forterre et al. 2014a, Van De Vlekkert et al. 2020, Wu et al. 2023). In addition, Calnexin, frequently used as an indicator that vesicles may be of endoplasmic reticulum origin, was negative for both EV samples, while a band was observed in the cell lysate sample (unpublished results). We used Follistatin as a protein expected to be at elevated levels in myoblast cell lysates and also to confirm the cell-type origin of the EV samples. Follistatin was indeed high in myoblast lysates and faintly visible in only the myo-EV sample while it was completely absent in the fibro-EV samples. SEM confirmed the isolation of small EVs, that were smaller than 200 nm. These results followed the MISEV 2018 and 2023 guidelines for the characterisation of small EVs (Théry et al. 2018; Welsh et al. 2024).

In general, we showed that myoblasts preferentially take up myo-EVs and that EV depletion of FBS reduced control myoblast proliferation and differentiation (indicated via the expression of MRFs). Neither myo-EVs nor fibro-EVs under such EV-free culture conditions, could rescue proliferation and differentiation to more closely align to that seen in EV containing NGM. Myo- and fibro-EVs did however, have differential functional effects on recipient myoblast migration behavior. Fibro-EVs, but not myo-EVs, slowed down the rate of scratch closure as a result of decreased persistence of myoblast migration.

In this study, the ability of EVs from two different donor cell types to be internalised by C2C12 myoblasts was compared. We verified myo-EV uptake by recipient myoblasts within 5 h. The PKH67 puncta observed were similar to those presented by Luo et al. (2021). In addition, we illustrate that the labelled EVs tended to be found in the perinuclear region. This is similar to what has been found by de Gasperi et al. (2017) for small EVs harvested from cultured muscle fibre segments and added to proliferating myoblasts. Both myo- and fibro-EVs were efficiently internalised by the myoblasts. We observed a distinct earlier preference for myo-EVs’ uptake, already evident at 5 h, but not for fibro-EVs. At 48 h there was substantially more myo-EV uptake following an additional treatment at 24 h. The uptake of fibro-EVs improved at 48 h although not to the same extent as the uptake of myo-EVs. These findings highlight the potential for cell-specific interactions between EVs and their target cells.

It is now known that FBS used in culture media contain EVs that may affect cell activity (Aswad et al. 2016). Sawada et al. (2022) determined that EVs isolated from FBS and applied to C2C12 cells, inhibited their differentiation. A similar outcome was achieved by Aswad et al. (2016) using EV-depleted FBS in the culture media. Therefore, prior to any experimental work in the current study, FBS to be used as a supplement in culture media was depleted of interfering small particles. The 85% reduction in particle count in EV-DM compared to NGM was similar to that of commercially available EV-depleted media. This ensured minimal interference from such contaminating particles in subsequent small EV populations isolated from conditioned media harvested from myoblast or fibroblast cultures. Also, the experimental study design included two control groups: NGM which contained normal FBS and EV-DM containing FBS without EVs to mimic the cell culture conditions of the 2 experimental groups prior to adding the myo- or fibro-EVs. With this study design we could ascertain the effect of EV-depletion from the media separately from the effect of any exogenous EVs added with the background of EV-DM.

Previous studies have employed EVs generated by bone marrow-derived mesenchymal stem cells (MSCs) to investigate their effects on muscle regeneration in vivo (Gangadaran et al. 2017; Iyer et al. 2020; Nakamura et al. 2015). The EVs were applied in rodent models of muscle damaged by muscle lengthening contractions resulting in strain-induced injury (Iyer et al. 2020), femoral artery occlusion resulting in ischemic injury (Gangadaran et al. 2017) or cardiotoxin injection resulting in diffuse damage (Nakamura et al. 2015).

Iyer et al (2020) showed that regeneration could be improved within two weeks after lengthening contraction-induced injury of rat tibialis anterior with EVs having been injected into the muscle immediately after contractions as well as on day 5 and day 10. The primary effect was a significant increase in the number of multinucleated fibres suggesting an effect on myoblast fusion. Gangadaran et al (2017) showed that, with twice weekly EV treatment after ischemic injury, muscle perfusion was gradually restored to control levels over 3 weeks concomitant with improved capillarisation. This suggests that despite local application of EVs they have a systemic effect which might improve muscle regeneration. Similarly, Nakamura et al. (2015) gave intramuscular EV injections on days 1, 3 and 5 after cardiotoxin injury of mouse tibialis anterior muscle. Muscle regeneration was enhanced after 7 days as seen by significantly more centrally nucleated myofibres, greater capillarisation and reduced fibrosis, suggesting multiple modes of action. However, Iyer et al. (2020) observed different effects depending on the source of the EVs used for treatment. For example, Myogenin gene expression increased with administration of EVs derived from platelet rich plasma, but not with bone marrow MSC derived EVs. Therefore, the source of EVs seem to be important for the downstream effects.

In vitro experiments more specific to skeletal muscle have determined that EVs from differentiated C2C12 myotubes induced differentiation of C2C12 myoblasts into myotubes (Guescini et al. 2017; Forterre et al. 2014a, 2014b), while myoblast proliferation was simultaneously reduced (Forterre et al. 2014a, 2014b) (Table 2). These findings suggest that myotube-derived EVs carried pro-differentiation factors. An opposite trend was recorded when C2C12 myotubes were cultured under compromising conditions. EVs collected from myotubes cultured under inflammatory conditions inhibited myoblast differentiation to myotubes (Kim et al. 2018). Also, EVs collected under hypoxic conditions (Guescini et al. 2017), increased myoblast proliferation.

**Table 2:** A summary of the range of EV particle concentrations and sizes presented in previous studies. EVs were collected from culture media conditioned by C2C12 myoblasts or myotubes or primary cultured human skeletal myotubes

Reference	EV source	Particle/EV information	Application & EV concentration
(Forterre et al. 2014a)	EVs secreted from differentiated C2C12 myotubes	All particles < 500 nm No EV yield reported	Applied to C2C12 myoblasts for both proliferation and differentiation: - 2 µg/ml
(Forterre et al. 2014b)	EVs secreted from C2C12 myoblasts and myotubes	Average EV size: 50–150 nm Particle yield: From myoblasts: 0.37 ± 0.15/ml/24 h From myotubes: 0.41 ± 0.23/ml/24 h	Applied to C2C12 myoblasts: - 2 µg control or transfected EVs
(Choi et al. 2016)	EVs secreted from differentiated primary cultured human skeletal myotubes (HSkM)	Mean EV size: 113.7 nm Particle yield: From myotubes: 1.7 × 10^9^ EV/μg protein	Range of EVs/ml applied: 10, 25, 50, 100, 200 Applied to human adipose-derived stem cells: - 50 µg protein/ml (optimal) *(= *8.5* × *10*^*10*^* EVs/ml)* Applied to in vivo injury: - 100 μg/100 μL PBS (optimal) *(= *1.7* × *10*^*11*^* EVs/100ul)*
(Guescini et al. 2017)	EVs secreted from control and oxygen stressed/hypoxic C2C12 myotubes	Mean EV size: 99 ± 11 nm No particle yield information	Range of EVs applied to C2C12 myoblasts: - 1 × 10^10^ and 5 × 10^10^ particles/ml
(Ji et al. 2022)	EVs secreted from C2C12 myotubes	Mean EV size: 139.1 nm Particle yield: From myotubes 6.4 × 10^10^ particles/ml	Applied to injured mouse skeletal muscle - 3.2 × 10^9^ EVs in 50 µl per injection every 2 days

*Calculations for further clarification are in italics

Since myoblasts are the main cell type contributing to skeletal muscle regeneration, the current study chose to harvest EVs from proliferating myoblasts instead of myotubes, representing an early phase of regeneration prior to myotube differentiation. Indeed, previously, significant differences in protein expression profiles of EVs from myoblast or differentiated myotubes were illustrated by Forterre et al. (2014a). It is also important to consider the culture conditions under which EVs have been collected. Sawada et al. (2022) showed that exosomes endogeneous to FBS are capable of exerting an inhibitive effect on the differentiation of C2C12 myogenic cells in vitro. For this reason, the current study used two control groups namely culture with complete FBS and culture with FBS depleted of EVs. We confirm that culturing myoblasts in EV-DM as opposed to NGM containing complete FBS significantly altered myoblast proliferation and Myogenin expression in the direction of enhanced differentiation. Treatment with EVs isolated from either myoblasts or fibroblasts also reduced proliferation and enhanced Myogenin expression when compared to the NGM condition. On the other hand, these effects of EV treatment were similar in extent from that seen in the EV-DM control condition, suggesting the strong effects of treatment was due to the elimination of EVs from FBS. There are two possible explanations for this observation. Firstly, the EVs in FBS may differ substantially from those that are secreted by myoblasts of fibroblasts. Secondly, the treatment concentration was too low for an independent effect and could not rescue the impact of EV depletion in the media.

We were aware of the discrepancy between the vesicle concentration and related protein concentrations in our samples. The range of EV particle concentrations used in previously published articles was summarized in Table 2. We used a treatment concentration of 2 × 10⁸ EVs/mL that was at the lower end of this range and that reflects physiological EV levels in human plasma (Lovett et al. 2018). Although Guescini et al. (2017) applied EVs to myotubes at concentrations of either 1 × 10 or 5 × 10^10^ particles per mL, neither concentration had effects on either myosin heavy chain or Myogenin protein levels when compared to their serum free media control (Guescini et al. 2017). Two studies applied EV treatment at concentrations calculated from EV protein levels rather than EV numbers. Forterre et al. (2014a) applied EVs in vitro at 2 μg/mL and Kim et al. (2018) added 3.3 μg/mL. Our study found that the fibro-EV samples had a 2.5-fold higher protein concentration than those isolated from myoblasts at the same particle number. We applied the EV treatment based on EV concentration. Despite the difference in EV protein content, the effects of EVs from both sources was not different for proliferation or differentiation. It is possible that potential differences were masked by the strong influence of culturing in EV-DM. Therefore, despite maintaining FBS concentration at 10% to evaluate effects of EVs under proliferative conditions, premature differentiation was observed when FBS was depleted of EVs. This argument is supported by the findings of Aswad et al. (2016) who also showed elevated Myogenin expression under EV-depleted conditions. Moreover, EVs isolated from FBS and applied to myoblasts have been shown to inhibit differentiation (Sawada et al. 2022), further indicating that the removal of EVs from FBS may accelerate differentiation. In short, even though fibro-EVs did contain a higher protein concentration than myo-EVs at the same vesicle density, no differences in proliferation results were observed. It was only for the migration assay that speed and directionality differed. In future studies, when treating myoblasts using EVs derived from different cell sources, it may be necessary to match particle number and add at least one other group that matches EV protein concentrations. A unit that reflects the number of EVs in relation to protein concentration may alternatively be considered.

As summarised by Mendias (2017), fibroblast play an important role in muscle development and regeneration outside of providing ECM. Using genetic ablation models Murphy et al. (2011) provided evidence of interaction between satellite cells and fibroblasts. Specifically, ablation of fibroblasts led to a reduced satellite cell pool size, premature differentiation and smaller myofibres after in vivo injury induced by cardiotoxin injection. Mackey et al. (2017) showed that in vitro culture of primary myoblasts and fibroblasts in direct contact stimulated proliferation only moderately but strongly stimulated differentiation and fusion in the presence of low horse serum. Mackey et al. (2017) also found that the separation of myoblasts and fibroblasts by a permeable membrane nullified any effect. This suggests that secreted factors were not involved.

To further explore the fibroblast/myoblast intercellular communication, we focussed on the effects of fibroblast-derived EVs from L929 cells on myoblasts. Indeed, in the context of wound healing, previous studies have indicated an effect of fibroblast derived EVs. EVs derived from primary dermal fibroblast culture accelerate wound healing in a diabetic rodent model in vivo (Han et al. 2021). Also, when EVs were isolated from immortalised L929 fibroblasts a positive effect on skin wound healing in a mouse model was observed (Oh et al. 2021). Similar to the model of Mackey et al. (2017) when fibroblast and myoblasts were separated by a permeable barrier, we found no effect of fibroblast derived EVs on proliferation or differentiation under EV-DM culture conditions. On the other hand, effects on myoblast migration were observed.

In contrast to the reduction in proliferation, we found that EV-DM increased the rate of scratch closure when compared to NGM. When myo-EVs were added there was an even greater improvement in scratch closure rate resulting in significant scratch closure at 10 h in that group. However, the addition of fibro-EVs slowed down the rate of closure of the scratch so that it did not differ from NGM but did differ from both EV-DM and myoEV groups. Also, the closure of the scratch at 10 h was highly variable. It is uncertain why this occurred, but it could be due to ECM components carried in fibroblasts EVs (Yeung et al. 2020).

The scratch assay results describe only the collective movement of cells at the leading front. The rate at which the front of a population of cells moves is dependent on the migration patterns of the individual cells. Therefore, the trajectories of individual cells were analysed in order to collect more specific information regarding either the randomness or persistence in the migratory pattern of these cells. The fibro-EVs’ effect on myoblasts resulted in the highest cumulative distance and speed of migration, and yet the linear displacement did not differ from the myo-EV treated myoblasts. This led to the persistence of the fibro-EV treated myoblasts in the forward direction proving to be significantly less than the myo-EV treated group and reflected and explains the poor scratch closure.

Cell migration is controlled by focal adhesions (FA) that form bridges between the intracellular actin network, integrins and their ligands in the ECM reviewed by Yamaguchi and Knaut (2022). While control over FA turnover regulates the speed of migrating myoblasts (Goetsch et al. 2014), digestion or remodelling of the ECM is also a vital factor. The importance of ECM-digesting matrix metalloproteases (MMPs), including the soluble MMP2 was illustrated in migrating C2C12 myoblasts (El Fahime et al. 2000).

EVs isolated from primary cultured skin fibroblasts also contained MMP2 (Gangadaran et al. 2022). Yeung et al. (2020) compared the protein cargo of myoblast and muscle fibroblast EVs using proteomics. Fibroblast EVs were highly significantly enriched in ECM-related proteins in particular fibulin-1 and nidogen-2, as well as MMP2 which is involved in reorganisation of the ECM. Other authors reported that corneal fibroblast EVs contain MMP1 and MMP2 and EVs enhanced epithelial cell migration; specifically, epithelial cells that internalised more fibroblast EVs migrated faster (Yeung et al. 2022). Oh et al. (2021) applied EVs isolated from L929 fibroblasts to migrating L929 cells in a scratch closure assay and the EVs promoted closure of the scratch. These authors further showed that the EV treated fibroblasts increased endogenous expression of MMP1 and MMP3 genes. Finally, Bodasa et al. (2023) illustrated the relevance of EV origin in affecting the function of target cells. Fibroblast EVs were collected from a Muscular Dystrophy patient that expressed a mutated collagen-VI. Treatment of control fibroblasts with EVs from either the patient or healthy fibroblasts increased cell migration speed and negatively affected directionality in the target cells, mimicking our results when fibro-EVs were added to myoblasts in a scratch assay.

Taking all these studies into consideration, it is possible that the fibro-EVs applied to myoblasts in the current study had an effect by providing ECM-related components and/or MMPs directly to the migratory environment or by altering gene expression of MMPs in the myoblasts.

In conclusion, mesenchymal stem cell-derived EVs have mainly been used in pre-clinical trials to assess potential therapeutic value (Rezaie et al. 2022). However, elucidating the effects of EVs derived from the cell types in the muscle niche which are involved in regenerative myogenesis might offer additional insights. By demonstrating that myoblasts preferentially internalise myo- over fibro-EVs, we reveal a cell-specific mechanism of EV uptake that has significant implications for muscle regeneration and repair. Our findings show that while myo-EVs enhance myoblast migration and directionality, fibro-EVs impair migration persistence. This suggests that EVs derived from different cell types within the muscle niche can exert distinct, even opposing, effects on cellular behaviour. The broader conclusions of this study are that the origin of EVs must be taken into account when considering specific outcomes, especially motility, and that preferential uptake of same cell-type-derived EVs does exist, at least for myoblasts. The biocompatibility and cell targeting capabilities of EVs shows great potential to revolutionized medicine and drug delivery (Chen et al. 2025). Future studies should ensure that cultures to be used for cell specific EV production are enriched in the selected cell type, containing no contaminating cells. This would be especially relevant when tissue explants are used to generate cell cultures for EV-related treatment.

## Data Availability

Data is provided within the manuscript text and figures and legends.
